# The Skin as an Indicator

**Published:** 1889-03-30

**Authors:** 


### THE SKIN AS AN INDICATOR.

To many persons disease of any kind is a profound mystery, and treatment is still more incomprehensible. The great advantage which the skin possesses when diseased is that the process can be seen by the patient as well as by the doctor, and that the result of treatment can also be witnessed. Not long ago, apatient who had a profusely discharging eczema, extending from the knee to the heel, was treated in a variety of ways without any good result. Week after week went by, and the leg showed no sign of general improvement whatever. At last an experienced doctor took the case in hand, and in a few days a marked change was evident. In a fortnight the worst symptoms had disappeared. In four or five weeks the case was quite well. The patient herself, and all her friends, as well as the doctor, were happy, because they could see the steady progress of the case from day to day. "Surely," says an eminent surgeon, "it cannot need much thought to perceive that the skin, inasmuch as it is exposed to view, affords the best opportunities for the inspection of pathological processes. The kinds of morbid action which it displays are almost infinitely varied, and they occur with a richness of combination that has not its parallel in any other organ. Diseases of the skin are invaluable, as helping us to the comprehension of pathological laws. . . . . They, better than any others, illustrate the influence of drugs upon the economy, and the marvellous manner in which idiosyncrasy controls such influence. We note with wonderment how, in the case of the iodides and bromides, the dose has little or no influence on the results; how very small doses will cure, and very large ones will not poison; and how, in the production of eruptions on the skin, all seems to depend upon peculiarity in the recipient, and nothing upon the quantity swallowed. Thus it has happened that the same patient has on three or four different occasions been poisoned, brought absolutely to death's door, by a dose of iodide of potassium, which most persons would have taken without knowing it. . . . There is, as I have said often before, no single fact in the range of therapeutics better calculated to rehabilitate our faith in drugs than to observe the manner in which a few drops of a tasteless solution of arsenic can dismiss an eruption of pemphigus, and rapidly restore the patient to health. . . . Another not unimportant pathological fact in reference to arsenic is the production of discolorations and chronic inflammations of the skin, ending in the formation of corns, which have a tendency to pass into epithelial cancer." This truly philosophical but simple illustration of general principles is of first-rate importance both to medical men and their patients: to medical men, as showing them the potency of the tools with which they work, and the necessity for a wide and accurate knowledge of their physiological and therapeutic effects; and to patients as demonstrating in a most convincing manner the absurdity and futility of what is known as "self-medication," when there is anything really the matter.

